# UPLC-MS/MS-based metabolomics analysis identifies disease-associated metabolic signatures of B cells from patients with IgG4-RD

**DOI:** 10.3389/fimmu.2026.1777700

**Published:** 2026-04-01

**Authors:** Xing Ji, Pingying Qing, Hanqiu Lyu, Sijun Zhang, Yinlan Wu, Yuqing Zhuang, Yanhong Li, Zhigang Tang, Chunyu Tan, Yubin Luo

**Affiliations:** 1 Department of Rheumatology and Immunology, West China Hospital, Sichuan University, Chengdu, Sichuan, China; 2 Frontiers Science Center for Disease-related Molecular Network, West China Hospital, Sichuan University, Chengdu, Sichuan, China; 3 Key Laboratory of Drug-Targeting and Drug Delivery System of the Education Ministry and Sichuan Province, Department of Medicinal Chemistry, West China School of Pharmacy, Sichuan University, Chengdu, Sichuan, China

**Keywords:** B cell, IgG4 - related disease, lipid metabolism, metabolomics, plasmablast

## Abstract

**Background:**

Immunoglobulin G4-related disease (IgG4-RD) is an autoimmune-mediated fibro-inflammatory disorder. Enhanced B-cell differentiation, along with its contribution to inflammatory and fibrotic pathological processes, represents a core mechanism in IgG4-RD. However, the metabolic characteristics of B cells in these patients have yet to be systematically investigated.

**Methods:**

Targeted metabolomic analysis was performed on sorted B cells from 32 IgG4-RD patients and 31 healthy controls using ultra-performance liquid chromatography-tandem mass spectrometry (UPLC-MS/MS). Differential metabolites between the two groups were identified using the Mann-Whitney U test and orthogonal partial least squares-discriminant analysis (OPLS-DA). Metabolic pathway enrichment analysis of differentially expressed metabolites was conducted using the MetaboAnalyst 6.0 platform. In an independent validation cohort, flow cytometry was employed to detect B-cell subpopulations, lipid content, and the expression of stearoyl-CoA desaturase 1 (SCD1).

**Results:**

Significant differences in metabolite profiles were observed between B cells from IgG4-RD patients and healthy controls. Compared to healthy controls, 24 metabolites were significantly upregulated and 124 metabolites were significantly downregulated in B cells from IgG4-RD patients. A panel of 14 metabolites demonstrated optimal performance in distinguishing IgG4-RD B cells from control B cells (AUC = 0.934). The upregulated metabolites were primarily enriched in pathways such as glutathione metabolism and unsaturated fatty acid biosynthesis, whereas various amino acid metabolism pathways were significantly downregulated. Lipid content was significantly elevated in B cells of IgG4-RD patients compared to healthy controls, with plasmablasts/plasma cells exhibiting the highest lipid content and fatty acid synthase expression among all B-cell subsets. The glutamate/glutamine ratio was significantly elevated in B cells of the fibrotic subgroup compared to the inflammatory subgroup of IgG4-RD. Taurocholic acid (TCA) and dimethylglycine showed correlations with serum IgG4 and IgE levels, respectively.

**Conclusion:**

This study reveals distinct metabolic features of pathogenic B cells in IgG4-RD, proposing the hypothesis that metabolic dysregulation contributes to their pathogenic alterations.

## 1 Introduction

IgG4-related disease (IgG4-RD) is a systemic fibro-inflammatory disorder associated with substantial medical and economic burden (1, 2). Elevated serum IgG4 levels and oligoclonal expansion of plasmablasts and plasma cells in affected tissues point to an autoimmune mechanism driving IgG4-RD (3, 4). The promising efficacy of B cell-targeted therapies—such as rituximab (5), inebilizumab (6), obexelimab (7), and Bruton’s tyrosine kinase inhibitors—further underscores the central role of B cells in the pathogenesis of IgG4-RD. Studies have shown that B cells in IgG4-RD patients not only contribute to inflammation via the IL-1β pathway (8), but also promote tissue fibrosis through the secretion of platelet-derived growth factor B (9). Thus, elucidating the phenotypic and functional alterations of B cells in IgG4-RD and uncovering the regulatory mechanisms involved are critical for advancing our understanding of the disease and developing more targeted therapeutic interventions.

Metabolites are downstream products of intracellular biochemical reactions. The analysis of metabolite alterations through metabolomics provides a unique perspective for both disease diagnosis and mechanistic investigation. Recent studies have emphasized the potential of metabolites as biomarkers for diagnosing and differentiating IgG4-RD. Notably, several characteristic metabolic signatures of IgG4-RD have been identified, including various serum metabolites such as polyunsaturated fatty acids, glutamate, and phospholipids, along with tissue-specific accumulation of arachidonic acid and enriched fecal bile acid metabolic pathways (10–14). However, these investigations remain constrained by limited sample diversity and have not yet comprehensively explored the metabolic profiles of pathogenic immune cells, leaving an incomplete understanding of IgG4-RD pathogenesis.

Advances in immunometabolism have established that metabolic reprogramming orchestrates B cell development, activation, and differentiation (15). The dysregulation of these pathways is increasingly implicated in the pathogenesis of autoimmune diseases—including systemic lupus erythematosus (16–18), rheumatoid arthritis (19), and multiple sclerosis (20, 21)—and has emerged as a key therapeutic target. In contrast, the role of B cell metabolism in IgG4-RD remains largely unexplored. In this study, we utilized ultra-performance liquid chromatography–tandem mass spectrometry (UPLC–MS/MS) to perform targeted metabolomic profiling of sorted B cells from patients with IgG4-RD. This approach aimed to comprehensively characterize the metabolic landscape of pathogenic B cells and to identify altered metabolic pathways that may contribute to their dysregulated immune function. Our findings provide novel insights that may facilitate the discovery of new B cell-specific therapeutic targets.

## 2 Methods

### 2.1 Patients and samples

This study was approved by the Ethics Committee of West China Hospital, Sichuan University and was conducted in accordance with the principles of the Declaration of Helsinki. Written informed consent was obtained from all participants prior to enrollment. A total of 32 patients who met the 2019 American College of Rheumatology/European League Against Rheumatism classification criteria for IgG4-related disease (IgG4-RD) were included, among whom 9 had never received steroids or immunosuppressants. Additionally, 31 age- and sex-matched healthy controls were recruited from the health examination center. The baseline characteristics of the two groups are presented in **Supplementary Table 1**.

Each participant underwent an overnight fast of at least 8 hours, after which a peripheral venous blood sample of approximately 10 mL was obtained. Within 3 hours after collection, fresh peripheral blood mononuclear cells (PBMCs) were isolated using Ficoll density gradient centrifugation. To ensure the stability of the cellular metabolomic profiles, most steps were performed at low temperature (4 °C). CD19^+^ B cells were then purified from the PBMCs using the EasySep™ Human B Cell Isolation Kit (STEMCELL Technologies, Cat. #17754). Cell viability was assessed by trypan blue staining, ensuring a viability rate greater than 95%. The cells were then counted and immediately stored at -80 °C for subsequent analysis. Throughout the experiment, all samples were strictly subjected to only one freeze-thaw cycle to avoid the effects of repeated freezing and thawing on metabolites. The maximum storage duration of cells at -80 °C did not exceed 6 months, and the detailed storage period for each sample was recorded prior to analysis to ensure the reliability and reproducibility of the metabolomic data.

### 2.2 Targeted metabolomic analysis

#### 2.2.1 Sample preparation

All cell samples were rapidly thawed on ice. Each sample was supplemented with ten pre-chilled zirconium oxide beads and 20 µL of deionized water, followed by homogenization for 3 minutes. Metabolite extraction was performed by adding 150 μL of internal standard solution in methanol, after which the samples were mixed for an additional 3 minutes and centrifuged at 18,000 g for 20 minutes. The supernatant was carefully collected and transferred to a 96-well plate. Concurrently, a 10 μL aliquot of the supernatant from each sample was taken and combined with 300 μL of methanol containing 5 mM ammonium acetate for lipid extraction. These mixtures were vortexed for 3 minutes and centrifuged again at 18,000 g for 20 minutes. Then, 20 μL of the resulting supernatant was transferred to a new 96-well plate and diluted with 80 μL of methanol containing 5 mM ammonium acetate. Both 96-well plates were sealed and prepared for LC-MS analysis.

#### 2.2.2 Instrumental analysis

This study employed an Ultra-Performance Liquid Chromatography-Tandem Mass Spectrometry (UPLC-MS/MS) system, specifically the ACQUITY UPLC-Xevo TQ-S model from Waters Corp. (Milford, MA, USA), for the targeted quantitative analysis of 600 metabolites, including amino acids, organic acids, fatty acids, carbohydrates, among others. The chromatography system utilized an ACQUITY UPLC BEH C18 1.7µM guard column (2.1 × 5 mm) and an ACQUITY UPLC BEH C18 1.7µM analytical column (2.1 × 100 mm), maintained at a column temperature of 40 °C. The ion source temperature was set at 150 °C, the desolvation temperature at 550 °C, and the desolvation gas flow rate at 1000 L/Hr. The differences in mobile phase composition, gradient elution program, flow rate, injection volume, and capillary voltage for the analysis of lipids versus other metabolites are detailed in **Table 1**.

**Table 1:** UPLC-MS/MS analysis parameters for lipids and other metabolites.

Parameter	Lipids	Other metabolites
Mobile Phase	A: Acetonitrile/Water (6:4, v/v) containing 5 mM ammonium formate and 0.1% formic acid. B: Isopropanol/Acetonitrile (9:1, v/v) containing 5 mM ammonium formate and 0.1% formic acid.	A: Water containing 0.1% formic acid. B: Acetonitrile/Isopropanol (70:30, v/v).
Gradient Elution Program	0–0.5 min (60% B), 0.5–3.0 min (60%→80% B), 3.0–7.0 min (80%→100% B), 7.0–9.0 min (100% B), 9.0–9.5 min (100%→60% B), 9.5–11.0 min (60% B).	0–1.0 min (5% B), 1.0–11.0 min (5%→78% B), 11.0–13.5 min (78%→95% B), 13.5–14.0 min (95%→100% B), 14.0–16.0 min (100% B), 16.0–16.1 min (100%→5% B), 16.1–18.0 min (5% B).
Flow Rate	0.3 mL/min	0.40 mL/min
Injection Volume	2 µL	5 µL
Capillary Voltage (kV)	3.0 (ESI^+^ mode)	1.5 (ESI^+^ mode), 2.0 (ESI^-^ mode)

#### 2.2.3 Quality control

During the analysis, three types of quality control samples (test mixture, internal standard, mixed biological sample) were prepared concurrently with the experimental samples and injected periodically throughout the analytical process. Blank samples were used to wash the chromatographic column, eliminate accumulated matrix effects, and confirm the absence of system contamination. The calibration set included a blank sample (matrix without internal standard), a zero sample (matrix with internal standard), and a series of seven concentration levels of standard solutions. Standard curves were constructed to ensure the accuracy and precision of the measurements.

### 2.3 Flow cytometry

For surface staining, cells were incubated at 4 °C for 30 minutes with the following antibodies: BV510-conjugated mouse anti-human CD19 (1:50; BD, Cat#: 562947), APC-Cy7-conjugated mouse anti-human CD20 (1:50; BD, Cat#: 302314), and BV605-conjugated mouse anti-human CD27 (1:50; BD, Cat#: 562655). For lipid staining, cells were treated with 500 µL of BODIPY 493/503 (10 µM, Thermo Fisher Scientific, Cat#: D3922) per tube and incubated at 4 °C for 30 minutes in the dark. For intracellular staining, cells were fixed and permeabilized using the Invitrogen Fixation/Permeabilization Kit (Thermo Fisher Scientific, Cat#: 00-5521-00) and the accompanying buffer (Thermo Fisher Scientific, Cat#: 00-8333-56), according to the manufacturer’s protocol. Subsequently, cells were incubated with a primary antibody against SCD-1 (1:500; abcam, Cat#: ab236868) diluted in 50 µL permeabilization buffer per tube for 30 minutes at low temperature in the dark. Following a wash step, cells were incubated with a PE-conjugated donkey anti-rabbit IgG secondary antibody (1:1000; BioLegend, Cat#: 406421) under the same conditions. After final washing, cells were resuspended in 500 µL of permeabilization buffer and analyzed on a FACSCanto II flow cytometer (BD Biosciences).

### 2.4 Transcriptomic metabolic pathway enrichment analysis

To investigate the characteristics of metabolic reprogramming in B cells under disease conditions, this study conducted a metabolic activity analysis of B cells and their subsets from both the disease and control groups, based on previously obtained single-cell RNA sequencing data (22). First, gene expression data were extracted from the raw expression matrix for B cells and their major subsets. Subsequently, single-cell metabolic pathway scoring was performed using the scMetabolism package in R language (23). This tool integrates the KEGG database (85 pathways) and the Reactome database (82 pathways), enabling quantification of metabolic pathway activity in individual cells. During the analysis, the default VISION algorithm was employed to calculate enrichment scores for each metabolic pathway, inferring metabolic activity by evaluating the overall expression level of gene sets within the pathways. By comparing the differences in metabolic pathway scores between the disease and control groups, significantly dysregulated metabolic pathways were identified. To further clarify whether the alterations in metabolic pathways are subset-specific, the above analysis was also performed separately on each B cell subset in this study.

### 2.5 Data analysis

Raw data files acquired by UPLC-MS/MS were processed using TMBQ software (v1.0, Metabo Profile, Shanghai, China) to quantify metabolite expression levels in each sample. Statistical analyses were performed using GraphPad Prism 8 and R 4.4.1. Multivariate statistical approaches, including Principal Component Analysis (PCA) and Orthogonal Partial Least Squares-Discriminant Analysis (OPLS-DA), were employed to identify outliers, assess clustering patterns, detect categorical trends, and examine differences in metabolite profiles between sample subgroups. Flow cytometry data were analyzed using FlowJo software (v10.8, BD Biosciences). The proportions of each cell subset were determined through predefined gating strategies, and the expression levels of target proteins were quantified using mean fluorescence intensity. For intergroup comparisons, an independent samples t-test was used for data meeting assumptions of normality and homogeneity of variances; otherwise, the Mann-Whitney U test was applied (for two-group comparisons). The Mann-Whitney U test was also used to compare metabolite concentrations between the disease and control groups. Metabolites satisfying both Variable Importance in Projection (VIP) score >1 and p-value <0.05 were subjected to pathway analysis using MetaboAnalyst 6.0. The discriminatory ability of each metabolite was evaluated by the area under the receiver operating characteristic curve (AUC), and a logistic regression model was employed to select optimal combinations of metabolites with the best discriminative power. Associations between variables were assessed using Spearman correlation analysis. A two-sided α level of less than 0.05 was considered statistically significant.

## 3 Results

### 3.1 B cells from patients with IgG4-RD exhibit a different metabolomic composition compared to healthy individuals

**Figure 1:**
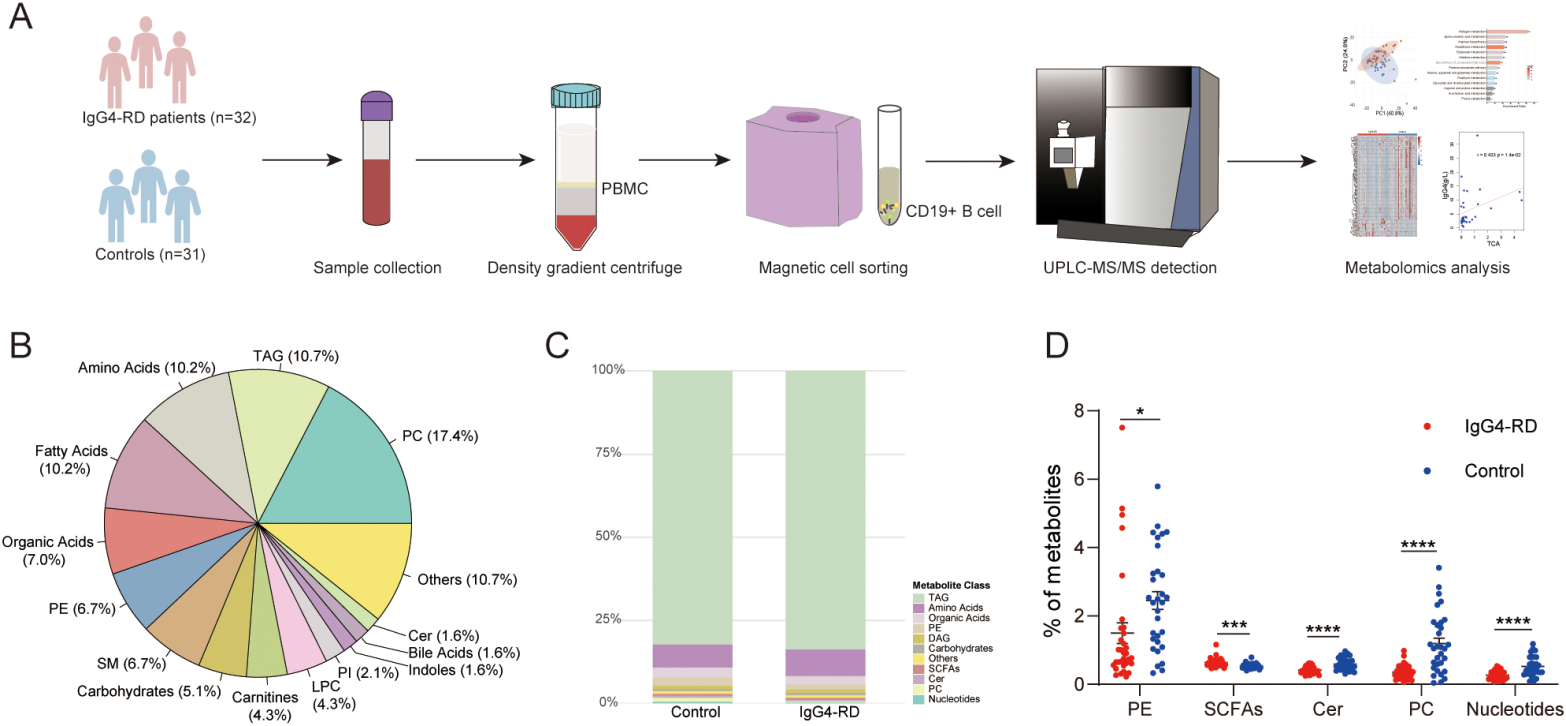
Summary of B-cell cohort study using ultra-high performance liquid chromatography and statistical analysis. **(A)** Workflow diagram of the present study. **(B)** Pie chart of the 15 chemical superclasses of 374 B-cell metabolites. **(C)** Bar graph showing the relative proportions of major metabolite types in B-cells from the HC and IgG4-RD groups based on metabolomics data. **(D)** Comparative analysis of the proportions of five metabolite classes between the HC and IgG4-RD groups. *P < 0.05, ***P < 0.001, ****P < 0.0001; PBMC, Peripheral Blood Mononuclear Cell; UPLC-MS/MS, Ultra-Performance Liquid Chromatography-Tandem Mass Spectrometry; TAG, Triacylglycerol; PC, Phosphatidylcholine; Cer, Ceramide; PI, Phosphatidylinositol; LPC, Lysophosphatidylcholine; SM, Sphingomyelin; PE, Phosphatidylethanolamine; DAG, Diacylglycerol; SCFAs, Short-Chain Fatty Acids.

To characterize the metabolomic features of B cells in IgG4-RD, CD19^+^ B cells were isolated from 32 patients and 31 healthy controls. Following isolation, cell viability (>95%) was confirmed by trypan blue staining, and precise cell counts were obtained using an automated counter. To account for variations in cell numbers, metabolite concentrations derived from targeted metabolomics were normalized to cell count (expressed as pmol per million cells). All 63 samples were then analyzed in a single batch using a well-established targeted metabolomics platform (**Figure 1A**). A mixture of authentic chemical standards and stable isotope-labeled internal standards was employed to ensure accurate metabolite quantification (24). The demographic characteristics, laboratory findings, affected organs, and treatment regimens of the patients are detailed in **Supplementary Tables 2** and **3**.

We detected a total of 374 metabolites in B cells and classified them into 28 categories. Lipid metabolites constituted the most abundant class, which included phosphatidylcholine (PC), triglycerides (TAG), fatty acids, phosphatidylethanolamine (PE), sphingomyelin (SM), and lysophosphatidylcholine (LPC). These were followed by amino acids, organic acids, and carbohydrates (**Figure 1B**). All these metabolites were present in both the disease and control groups, with TAG being the most abundant in B cells in both groups, although no statistical difference was observed (**Figure 1C**). Notably, short-chain fatty acids (SCFAs) were more abundant in the disease group than in the control group, whereas the levels of PE, PC, ceramide, and nucleotides were reduced compared to those in the control group (**Figure 1D**).

To evaluate the impact of treatment, we first performed partial least squares discriminant analysis (PLS-DA) to compare the metabolic profiles of B cells among the control, treated, and untreated groups. The results showed a clear trend of separation between the disease groups (including both treated and untreated patients) and the control group; however, notably, the metabolic profiles of patient samples from the treated and untreated groups largely overlapped (**Supplementary Figures 1A, B**). Subsequently, we screened for potential differential metabolites using a variable importance in projection (VIP) score greater than 1 as the threshold, combined with validation by the Mann-Whitney U test. No statistically significant differential metabolites were identified between the treated and untreated groups (**Supplementary Figure 1C**), suggesting that treatment may not substantially alter the B cell metabolome in IgG4-RD. Accordingly, treated and untreated patients were combined into a single disease group for subsequent comparisons with controls.

**Figure 2:**
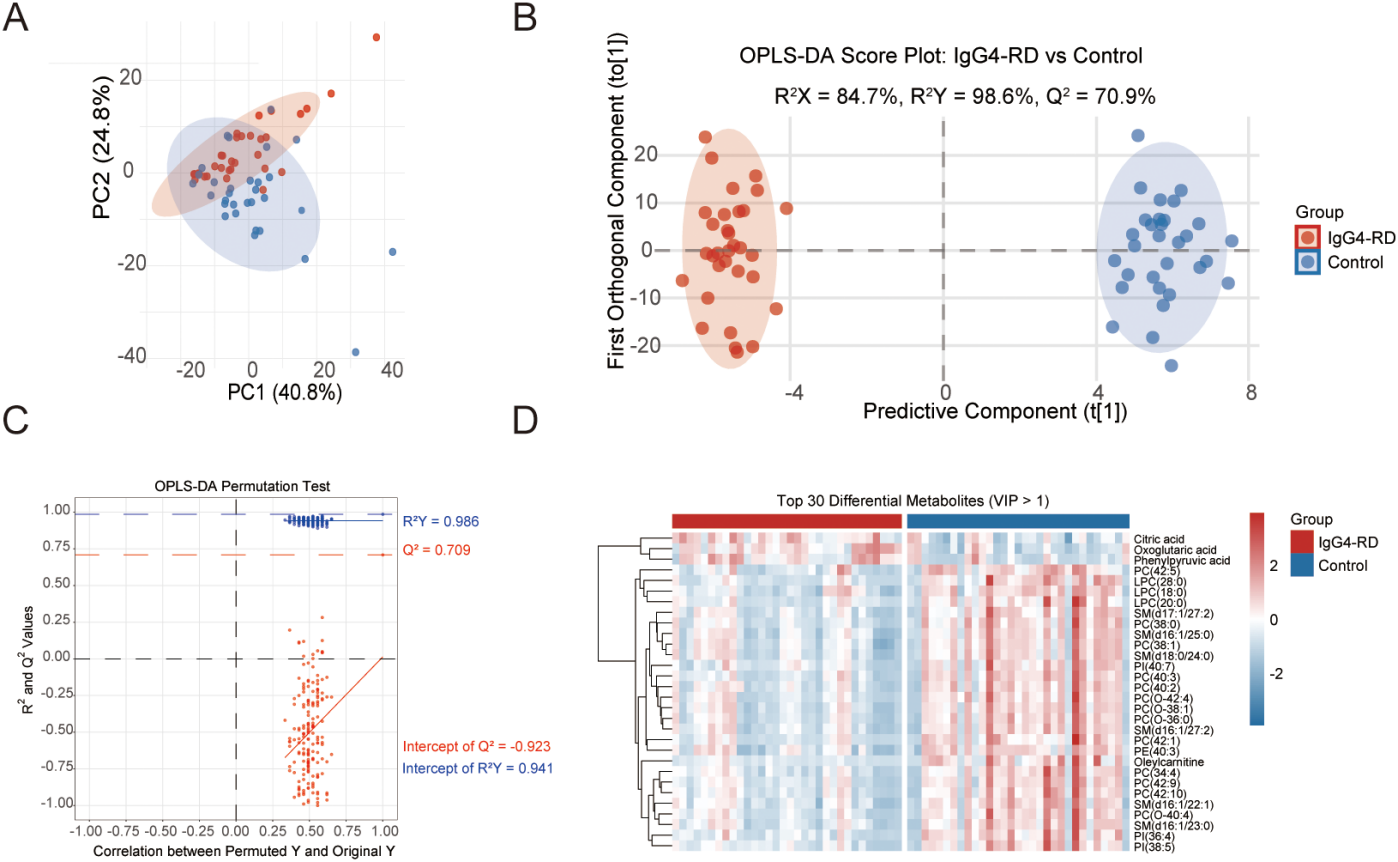
Multivariate analysis reveals significant differences in the B-cell metabolome between healthy controls and IgG4-RD patients. **(A)** PCA score plot of the B-cell metabolomic profiles from 32 IgG4-RD patients and 31 healthy controls (HCs). **(B)** OPLS-DA score plot of the B-cell metabolomic profiles from 32 IgG4-RD patients and 31 HCs. **(C)** The validation of the OPLS‐DA models from patients with IgG4-RD and HCs. **(D)**Heatmap of the top 30 discriminating features between HCs and IgG4-RD patients. PCA, principal component analysis; OPLS-DA, orthogonal partial least squares discriminant analysis; VIP, variable importance in projection.

To further investigate compositional differences between disease and control groups, principal component analysis (PCA) (**Figure 2A**) and orthogonal partial least squares discriminant analysis (OPLS-DA) were performed. OPLS-DA revealed a clearer separation than PCA, with high model reliability (R^2^X = 0.847, R^2^Y = 0.986, Q^2^ = 0.709) (**Figure 2B**). The validity of the OPLS-DA model was rigorously assessed using a permutation test. The resulting intercepts for R² and Q² were 0.941 and -0.923, respectively. These results confirm the model’s validity and the absence of overfitting, indicating that the observed group separation reflects genuine biological differences. A total of 152 metabolites had VIP > 1, indicating significant contributions to group discrimination. A heatmap of the top 30 metabolites confirmed distinct separation between groups (**Figure 2D**). Mann-Whitney U test identified 153 differentially abundant metabolites (FC > 1.2 or < 0.8, FDR < 0.05) (**Supplementary Figure 2A**). Integrating both approaches, we found 24 significantly elevated and 124 significantly reduced metabolites in IgG4-RD B cells compared to healthy controls (**Supplementary Figure 2B**). Among the differential metabolites, linoleylcarnitine, oleylcarnitine, LPC(28:0), tetradecanoylcarnitine, PC(42:2), CerPE(16:0), PC(42:10), and palmitoylcarnitine showed the most pronounced decreases, while gluconolactone, PE(32:1), oxoglutaric acid, palmitoleic acid, and methylcysteine exhibited the highest fold increases (**Supplementary Table 4**).

To further identify key discriminatory markers from these 148 differential metabolites, we selected 14 metabolites with individual areas under the curve (AUC) greater than 0.8 for distinguishing B cells between IgG4-RD patients and controls (**Supplementary Table 4**). PC(38:0), LPC(28:0), oxoglutaric acid, PI(36:4), PI(38:5), and PC(42:2) represented the top six candidates (**Supplementary Figure 3A**). We then developed a logistic regression model incorporating all 14 metabolites, which demonstrated a strikingly superior discriminatory capacity, achieving a substantially elevated AUC of 0.934 compared to any single metabolite (**Supplementary Figure 3B**).

### 3.2 Lipid metabolism pathways are up-regulated in B cells of IgG4-RD patients

**Figure 3:**
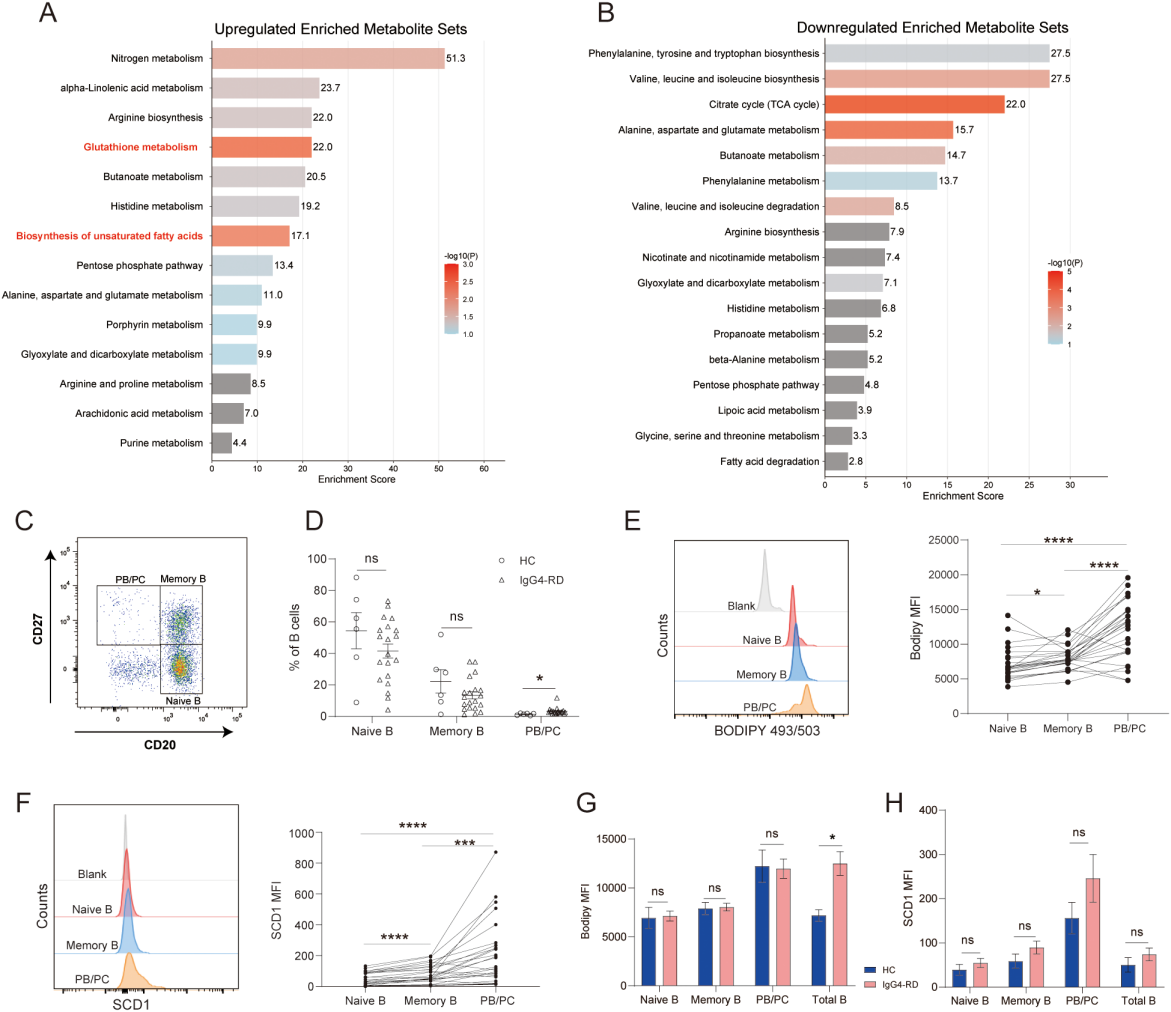
Lipid metabolic pathways are significantly up-regulated in B cells of IgG4-RD patients. **(A)** Bar graph showing the metabolite sets enriched by the 24 up-regulated metabolites. **(B)** Bar graph showing the metabolite sets enriched by the 124 down-regulated metabolites. **(C)** Gating strategy for naïve B cells, memory B cells, and plasmablasts/plasma cells. **(D)** Comparison of the proportions of the three B-cell subsets between IgG4-RD patients and HCs. **(E)** Quantitative comparison of lipid levels in the three B-cell subsets. **(F)** Quantitative comparison of SCD1 expression levels in the three B-cell subsets. **(G)** Comparison of lipid quantification in total B cells and the three subsets between IgG4-RD and HC group. **(H)** Comparison of SCD1 expression in total B cells and the three subsets between IgG4-RD and HC group. *P < 0.05, ***P < 0.001, ****P < 0.0001, ns: not significant; HC: healthy control; PB, plasmablast; PC, plasma cell; Stearoyl-CoA Desaturase 1.

Through pathway enrichment analysis of 148 differential metabolites, we sought to identify metabolic pathways potentially implicated in IgG4-RD pathogenesis. The results demonstrated marked upregulation of several lipid metabolism pathways in patient-derived B cells, encompassing alpha-linolenic acid metabolism, butanoate metabolism, biosynthesis of unsaturated fatty acids, and arachidonic acid metabolism. The glutathione metabolism pathway was also found to be enriched in B cells from IgG4-RD patients (**Figure 3A**). In contrast, downregulated metabolites were primarily associated with amino acid metabolic pathways, including the biosynthesis of phenylalanine, tyrosine, and tryptophan; valine, leucine, and isoleucine biosynthesis; and alanine, aspartate, and glutamate metabolism (**Figure 3B**). Within the glutathione metabolism pathway, both pyroglutamic acid and glutamic acid levels were elevated in IgG4-RD B cells relative to controls (**Supplementary Figure 4A**). Similarly, in the biosynthesis of unsaturated fatty acids pathway, alpha-linolenic acid and arachidonic acid were present at higher levels in B cells from the disease cohort (**Supplementary Figure 4B**).

To further validate our metabolomic findings, an independent cohort of 20 IgG4-RD patients and 6 healthy controls was recruited for comprehensive flow cytometric analysis (**Supplementary Tables 5**, **6**). Quantitative assessment of intracellular lipid levels in B-cell subsets was conducted using BODIPY 493/503 staining, and expression of stearoyl-CoA desaturase-1 (SCD1)—a pivotal enzyme in monounsaturated fatty acid synthesis—was systematically evaluated. Consistent with previous studies (4), IgG4-RD patients demonstrated a significantly expanded plasmablast/plasma cell compartment compared to controls (**Figures 3C, D**). Strikingly, this pathological cell population exhibited the most substantial lipid accumulation and highest SCD1 expression among all B-cell lineages (**Figures 3E, F**). However, intergroup comparisons revealed significantly elevated BODIPY fluorescence exclusively within the bulk CD19^+^ B-cell population from patients, corroborating our pathway analysis indicating activation of lipid biosynthetic pathways (**Figure 3G**). No statistically significant differences in SCD1 expression were observed across corresponding B-cell subpopulations between groups (**Figure 3H**). Collectively, the upregulation of lipid synthesis programs during plasmablast differentiation may contribute to the formation of pathogenic B cells in IgG4-RD.

We further analyzed our team’s previously generated single-cell transcriptomics data to compare metabolic pathway alterations at the transcriptomic level in B cells between IgG4-RD patients and healthy controls (22). Our analysis revealed upregulation of key energy metabolism pathways in patient-derived B cells, including Pyruvate Metabolism and Citric Acid (TCA) Cycle, Glutamate and Glutamine Metabolism, Glucose Metabolism, and Fatty Acid Metabolism (**Supplementary Figure 5A**). Notably, Pyruvate Metabolism and the TCA Cycle, Glutamate and Glutamine Metabolism, and Fatty Acid Metabolism showed the highest expression levels in plasma cells and differentiating plasmablasts (**Supplementary Figure 5B**). These findings suggest that enhanced energy metabolism may be one of the potential mechanisms driving B cell differentiation and contributing to the pathogenesis of IgG4-RD, a hypothesis that warrants validation through functional experiments in future studies.

### 3.3 A comparative analysis identified divergent B-cell metabolites between the clinical subtypes of IgG4-RD

**Figure 4:**
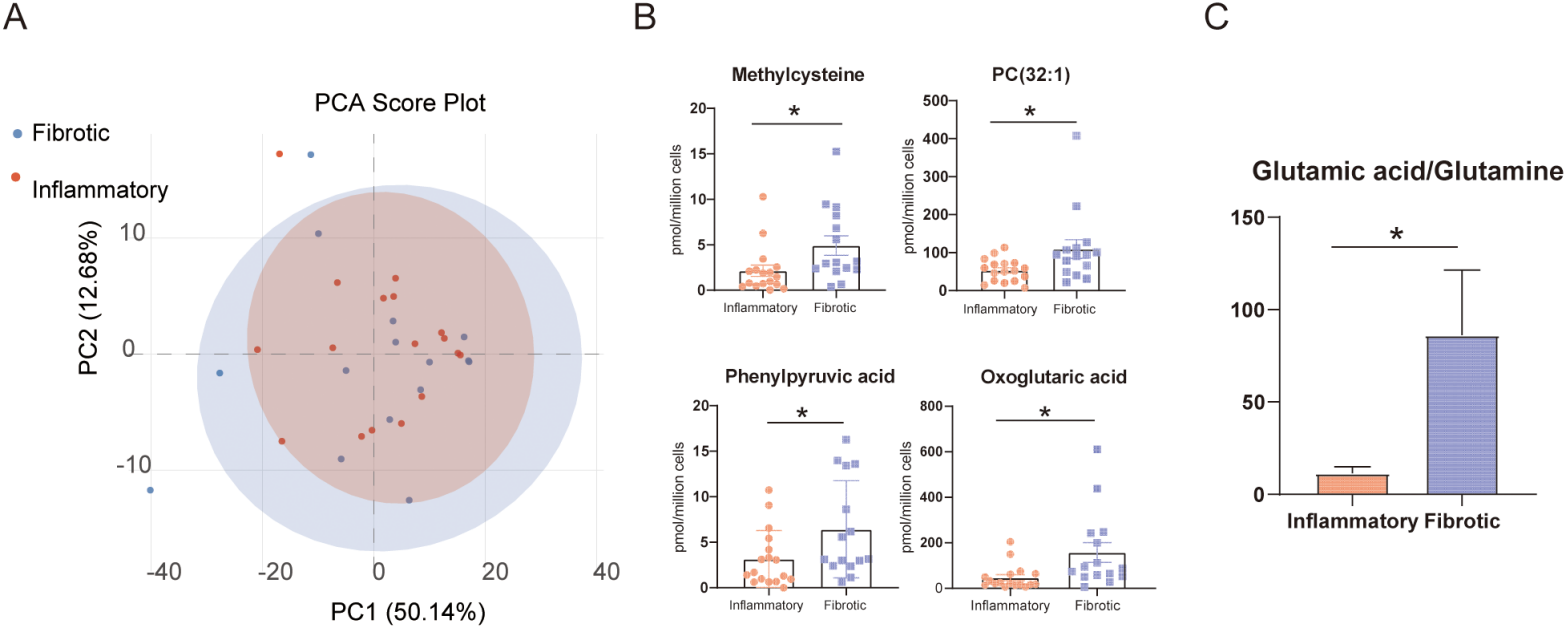
Comparative analysis identified divergent B-cell metabolites between the clinical subtypes of IgG4-RD. **(A)** PCA score plot of metabolomic profiles from 17 inflammatory-type IgG4-RD patients and 15 fibrotic-type IgG4-RD patients. **(B)** Four metabolites showing significant concentration differences between the two subgroups. **(C)** Comparison of glutamate-to-glutamine ratios between the inflammatory and fibrotic groups. *P<0.05.

B cells play a dual role in promoting both fibrosis and inflammation in IgG4-RD. A key question in current research is whether these distinct pathogenic functions are supported by different metabolic pathways. In this study, patients were classified into 17 inflammatory and 15 fibrotic subtypes according to organ involvement and pathological features (25, 26). The two groups were matched in terms of age and sex, and no significant statistical differences were observed in laboratory tests for IgG, IgG4, or IgE levels. The fibrotic subtype primarily involved tissues such as the retroperitoneum, skull base, and mediastinum, while the inflammatory subtype mainly affected the pancreas, lacrimal glands, biliary tract, and salivary glands. There were also significant differences in treatment regimens between the two groups, with glucocorticoid monotherapy being more common in the inflammatory subtype, while the fibrotic subtype was more inclined towards combination therapy with immunosuppressants (**Supplementary Table 7**). PCA of metabolomic data from the two groups showed extensive overlap in their metabolic profiles, with no clear separation trend (**Figure 4A**). Comparative t-test analysis between the groups identified four metabolites—oxoglutaric acid, methylcysteine, PC(32:1), and phenylpyruvic acid—that were elevated in the fibrotic subgroup (**Figure 4B**). Notably, although no significant differences were observed in glutamine or glutamate levels between the two groups, the glutamate/glutamine ratio was significantly elevated in the fibrotic subgroup (**Figure 4C**). These findings suggest an upregulation of glutamine catabolism in B cells of IgG4-RD patients with a fibrotic phenotype. Whether this represents a form of metabolic reprogramming that contributes to the pro-fibrotic effects of B cells warrants further investigation.

### 3.4 Metabolites in B cell are associated with disease activity and antibody production

**Figure 5:**
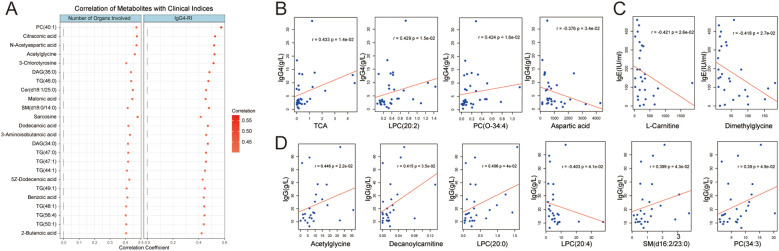
Analysis of correlations between B-cell metabolites and clinical indicators. **(A)** Top 24 B-cell metabolites showing positive correlations with number of involved organs and IgG4 response index. **(B)** Three B-cell metabolites positively correlated with serum IgG4 levels and one metabolite showing negative correlation. **(C)** Two B-cell metabolites negatively correlated with serum IgE levels. **(D)** Five B-cell metabolites positively correlated with serum IgG levels and one metabolite showing negative correlation. TCA: Taurocholic Acid.

B cells in IgG4-RD are also characterized by enhanced plasmablast differentiation and significantly increased antibody production capacity. In this study, we found that multiple lipid metabolites, including PC(40:1), DAG(36:0), TG(46:0), Cer(d18:1/25:0), and SM(d18:0/14:0); amino acids and their derivatives: citraconic acid, N-acetylaspartic acid, acetylglycine, 3-chlorotyrosine, sarcosine, and 3-aminoisobutanoic acid; as well as fatty acids: dodecanoic acid, 5Z-dodecenoic acid, and 2-butenoic acid, all showed positive correlations with the number of involved organs and the response index in IgG4-RD (**Figure 5A**). Aspartic acid, LPC(20:2), and taurocholic acid (TCA) were positively correlated with serum IgG4 antibody levels, while PC(O-34:4) was negatively correlated with IgG4 levels (**Figure 5B**). B cells from patients with elevated serum IgG4 levels showed higher levels of TCA and 3-Methyladipic acid compared to those with normal serum IgG4 levels (**Supplementary Figure 6A**). Similarly, dimethylglycine and L-carnitine were negatively correlated with serum IgE levels (**Figure 5C**), and B cells from patients with elevated serum IgE levels exhibited lower levels of dimethylglycine, γ-aminobutyric acid (GABA), and lysine metabolites (**Supplementary Figure 6B**). Decanoylcarnitine, SM(d16:2/23:0), LPC(20:0), acetylglycine, and PC(34:3) showed positive correlations with total serum IgG levels, whereas LPC(20:4) exhibited a negative correlation (**Figure 5D**). These metabolic changes and their relationship with the antibody-producing capacity of B cells require further investigation.

## 4 Discussion

Accumulating evidence indicates that dysregulated B-cell activation—characterized by enhanced differentiation, increased antibody secretion, and pro-inflammatory or pro-fibrotic functions—represents a central element in the pathogenesis of IgG4-related disease (IgG4-RD) (27, 28). This study presents the first targeted quantitative metabolomic analysis of purified B cells, revealing a significantly altered metabolic landscape in B cells from IgG4-RD patients. Compared with healthy controls, peripheral B cells from patients exhibited marked metabolomic alterations, with 24 metabolites upregulated and 124 downregulated. A set of 14 metabolites collectively enabled effective discrimination between B cells from the disease and control groups. By integrating flow cytometry and transcriptomic data, we identified multiple altered metabolic pathways. Notably, the glutathione metabolism pathway and the biosynthesis of unsaturated fatty acids pathway were significantly upregulated in B cells from the disease group. Further investigation revealed metabolic variations between different clinical subtypes and correlations between metabolite abundances and clinical disease activity scores or antibody titers. Collectively, our findings reveal an association between B-cell metabolic alterations and IgG4-RD disease progression, providing new clues for further exploration of the pathogenic mechanisms of B cells in this disease.

Metabolomic research on IgG4-RD remains relatively limited. Current studies primarily employ non-targeted metabolomic approaches, with analyses restricted to plasma, tissue, and fecal samples. Despite these limitations, this methodology has successfully identified a range of metabolic biomarkers with the potential to differentiate IgG4-RD from other conditions (10–14). Yan et al. recruited four cohorts, including healthy controls, IgG4-RD, pancreatic cancer, and Sjögren’s syndrome (SS), for untargeted liquid chromatography-tandem mass spectrometry metabolomic analysis of plasma samples. They identified caftaric acid, maltotetraose, d-glutamic acid, 1-stearoyl-2-arachidonoyl-sn-glycero-3-phosphoserine, and hydroxyproline as five valuable biomarkers (11). Gong et al. found that IgG4-RD affects the plasma metabolic profile, but through treatment, the metabolic state may shift toward normal (10). Shimizu et al. compared lesioned tissues from IgG4-related ophthalmic disease and orbital lymphoma, revealing unique enrichment of the arachidonic acid metabolism pathway (13). However, the clinical translational potential of these findings is substantially constrained by heterogeneity in control cohorts and a lack of critical external validation.

Metabolic alterations in B cells of IgG4-RD patients have been preliminarily explored, yet their metabolic status remains controversial. Single-cell transcriptomic analysis revealed significant enrichment of the “PI3K-Akt signaling pathway” in unswitched memory B cells and enhanced glycolysis/gluconeogenesis signaling pathways in plasma cells of IgG4-RD patients (29). Our previous studies also demonstrated marked enrichment of oxidative phosphorylation in IgG4-RD B cells, which we hypothesize provides energy support for enhanced protein synthesis (22). However, seahorse metabolic analysis showed that compared to B cells from healthy controls, IgG4-RD B cells exhibited reduced basal respiration, maximal respiration, and glycolytic capacity. Immunofluorescence staining further indicated decreased mitochondrial/BCR colocalization both before and after stimulation, while flow cytometry confirmed lower mitochondrial membrane potential and reduced intracellular reactive oxygen species (ROS) levels (8). These findings collectively suggest that B cells in IgG4-RD patients are in a state of metabolic suppression. Therefore, further comprehensive investigations are required to elucidate the metabolic reprogramming in IgG4-RD B cells.

The first key finding of our study underscores the importance of lipid metabolites and their associated metabolic pathways in B cells during the pathogenesis of IgG4-RD. Specifically, B cells from IgG4-RD patients exhibited increased abundance of short-chain fatty acids (SCFAs) and a marked elevation in oxalic acid—a dicarboxylic acid—compared with controls. It has been previously reported that SCFAs at low concentrations moderately promote class-switch DNA recombination (30). In contrast, at higher levels, intracellular SCFAs inhibit histone deacetylase activity, selectively upregulate specific miRNAs, and downregulate the expression of activation-induced cytidine deaminase (AID) and B lymphocyte-induced maturation protein-1 (Blimp-1). Consequently, these effects are likely to compromise the formation of plasma cells that produce class-switched and hypermutated antibodies (30–32). Based on our findings, we proposed that oxalic acid may function as an energetic substrate to facilitate antibody class switching in IgG4-RD B cells. Oxalic acid undergoes mitochondrial fatty acid oxidation to generate acetyl-CoA, thereby fueling the tricarboxylic acid (TCA) cycle for energy production. This promotive effect was consistently observed in B cells from both systemic lupus erythematosus (SLE) patients and lupus-prone NZM2328 mice. Importantly, *in vivo* inhibition of carnitine palmitoyltransferase 1 (CPT1), a rate-limiting enzyme in fatty acid oxidation, significantly ameliorated the lupus-like disease phenotype in mice (18). In IgG4-RD patients, the levels of monounsaturated fatty acid palmitoleic acid and polyunsaturated fatty acid arachidonic acid in B cells were significantly elevated compared to those in healthy controls. KEGG pathway analysis revealed upregulation of unsaturated fatty acid biosynthesis pathways in patient-derived B cells, which was further corroborated by BODIPY 493/503 flow cytometry showing increased lipid content in IgG4-RD B cells. Analysis of lipid content across B-cell subsets indicated that lipid accumulation intensifies during plasma cell differentiation. Notably, stearoyl-CoA desaturase 1 (SCD1), the rate-limiting enzyme in monounsaturated fatty acid synthesis, also exhibited highest expression in plasma cells. Zhang et al. reported that supplementation with monounsaturated fatty acids during *in vitro* LPS or anti-CD40 stimulation significantly enhanced plasma cell differentiation, while treatment with a specific SCD inhibitor substantially suppressed this process. Consistent with these findings, genetic deletion of Scd1 in mice resulted in significantly reduced absolute numbers and percentages of B220^int^CD138^+^ plasma cells in the spleen (33). Given the shared B-cell phenotypic alterations observed in IgG4-RD and SLE, these results suggested that targeting lipid synthesis and catabolic pathways in B cells may represent a potential therapeutic strategy for IgG4-RD.

In B cells derived from patients with IgG4-RD, a spectrum of amino acids and related metabolites—including glutamic acid, pyroglutamic acid, homoserine, 1-methylhistidine, ketoleucine, phenylpyruvic acid, methylcysteine, homocitrulline, 3-methyl-2-oxopentanoic acid, and oxoglutaric acid—was found to be upregulated. Notably, glutamic acid, pyroglutamic acid, and oxoglutaric acid are all closely associated with glutamine metabolism. Consistent with this observation, glutathione metabolism pathway was also upregulated, reinforcing the involvement of glutamine/glutamate-dependent metabolic reprogramming in B cells of IgG4-RD. It is well established that glutamine promotes the generation and function of IL-10-producing regulatory B cells through the mTOR/GSK3 pathway (34, 35). However, this study did not evaluate B10 cells in the context of IgG4-RD. Sumikawa et al. reported that both glutamine deprivation and inhibition of glutaminase with BPTES attenuated plasmablast differentiation (17). Further analysis demonstrated an elevated glutamate/glutamine ratio in B cells of fibrotic-subtype IgG4-RD patients relative to their inflammatory-subtype counterparts, implicating increased glutaminase (GLS) activity (36, 37). Whether this metabolic shift contributes to the pro-fibrotic behavior of B cells in IgG4-RD warrants further investigation.

This study has several limitations. The relatively small sample size limited our ability to perform more detailed stratification of IgG4-RD samples, for example, pre-treatment and post-treatment patients could not be individually matched. Additionally, the absence of an external B-cell validation cohort may raise concerns regarding the generalizability of the metabolomic findings. This study can only be regarded as a preliminary investigation into the relationship between B-cell metabolic reprogramming and the pathogenesis of IgG4-RD, and substantial, logically rigorous fundamental research is still required to fully elucidate this connection. Nevertheless, our study expands the current understanding of IgG4-RD by highlighting the potential of metabolomics in unraveling the complex metabolic alterations in B cells. These insights pave the way for exploring novel therapeutic targets and contribute to the advancement of IgG4-RD research and treatment.

## Data Availability

The raw data supporting the conclusions of this article will be made available by the authors, without undue reservation.
