# Supplementary material for: UPLC-MS/MS-based metabolomics analysis identifies disease-associated metabolic signatures of B cells from patients with IgG4-RD
Source: Front Immunol. 2026 Apr 1;17:1777700. doi: 10.3389/fimmu.2026.1777700 (PMC13079334; doi:10.3389/fimmu.2026.1777700)
Supplement: Supplementary file 1 [file DataSheet1.docx]

**Supplementary Figures**

**
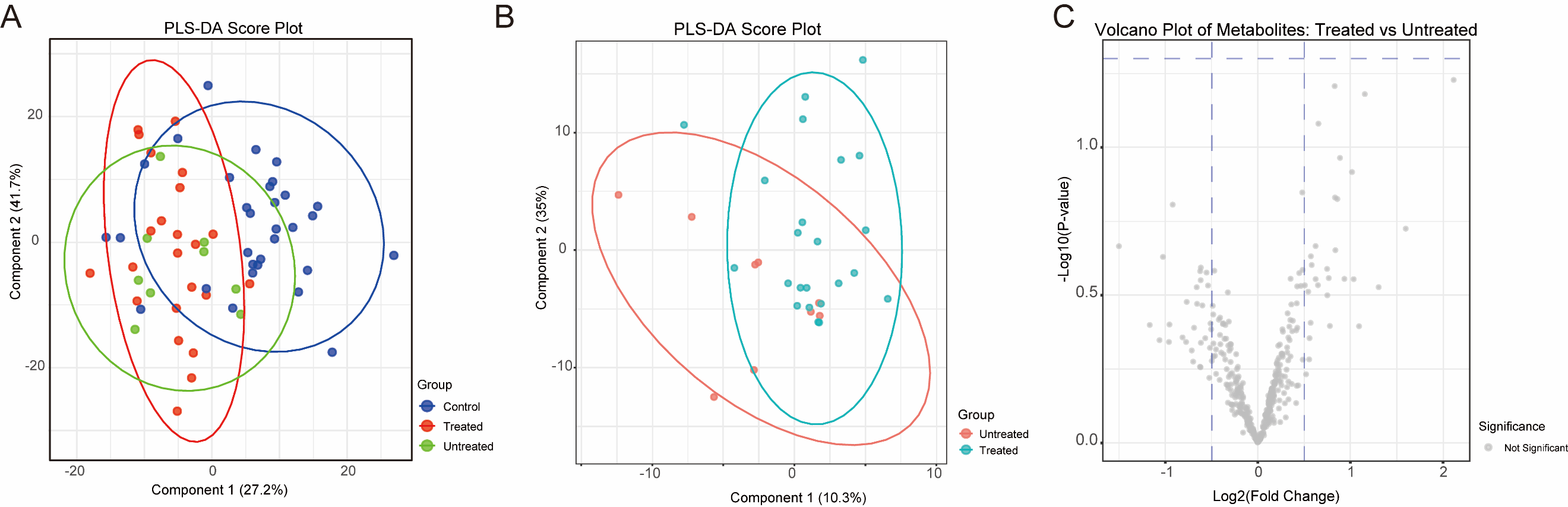
**

**Supplementary Figure 1. Metabolomic analysis of B cells from IgG4-RD patients and healthy controls**

**(**A) PLS-DA score plot of the B-cell metabolomic profiles from untreated IgG4-RD patients, treated IgG4-RD patients, and controls.
(B) PLS-DA score plot of the B-cell metabolomic profiles from 9 untreated IgG4-RD patients and 23 treated IgG4-RD patients.
(C) Volcano plot illustrating differential metabolites in B-cell metabolomic profiles of untreated IgG4-RD patients compared to treated IgG4-RD patients, analyzed using the Mann-Whitney U test.

PLS-DA, partial least squares discriminant analysis.


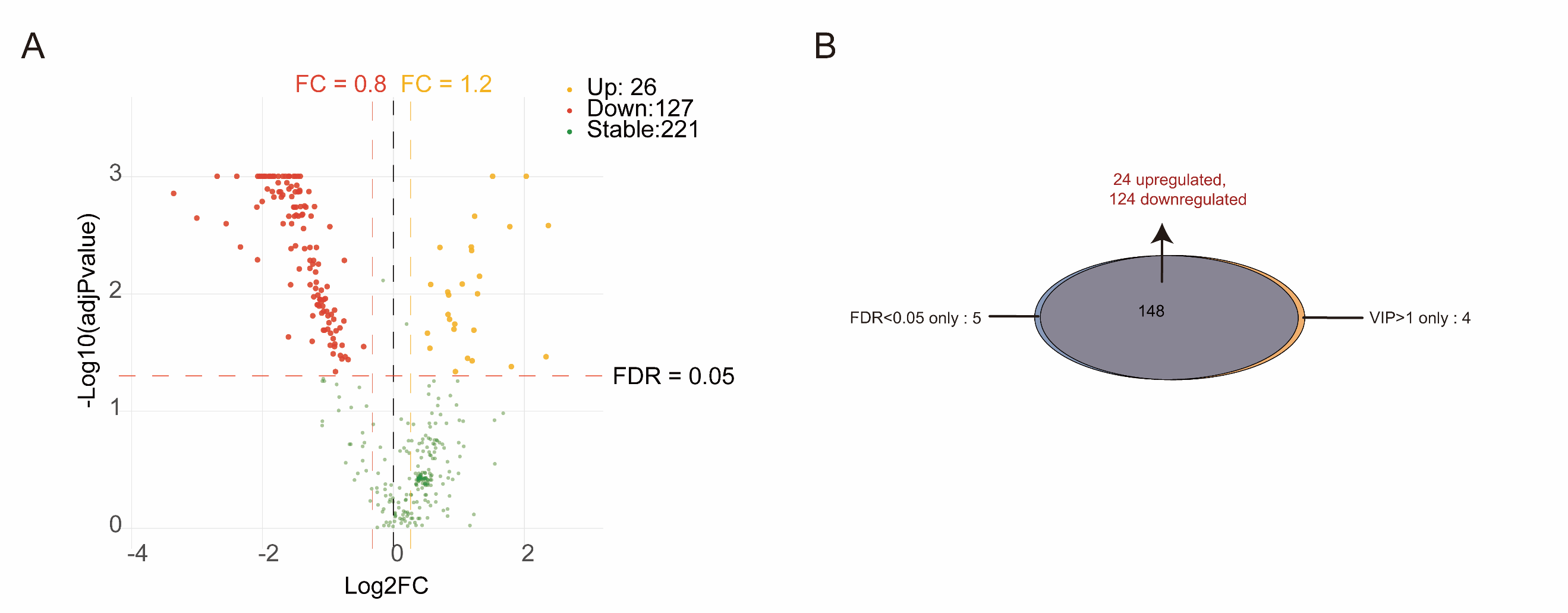


**Supplementary Figure 2. Univariate analysis reveals differential metabolites in B cells between healthy controls and IgG4-RD patients.**

(A) Volcano plot depicting differential metabolites identified by the Mann-Whitney U test in B cells from IgG4-RD patients versus HCs.
(B) Venn diagram illustrating the overlap between differential metabolites identified by univariate and multivariate analysis.

FC, fold change; FDR, false discovery rate; VIP, variable importance in projection; HC, healthy control.


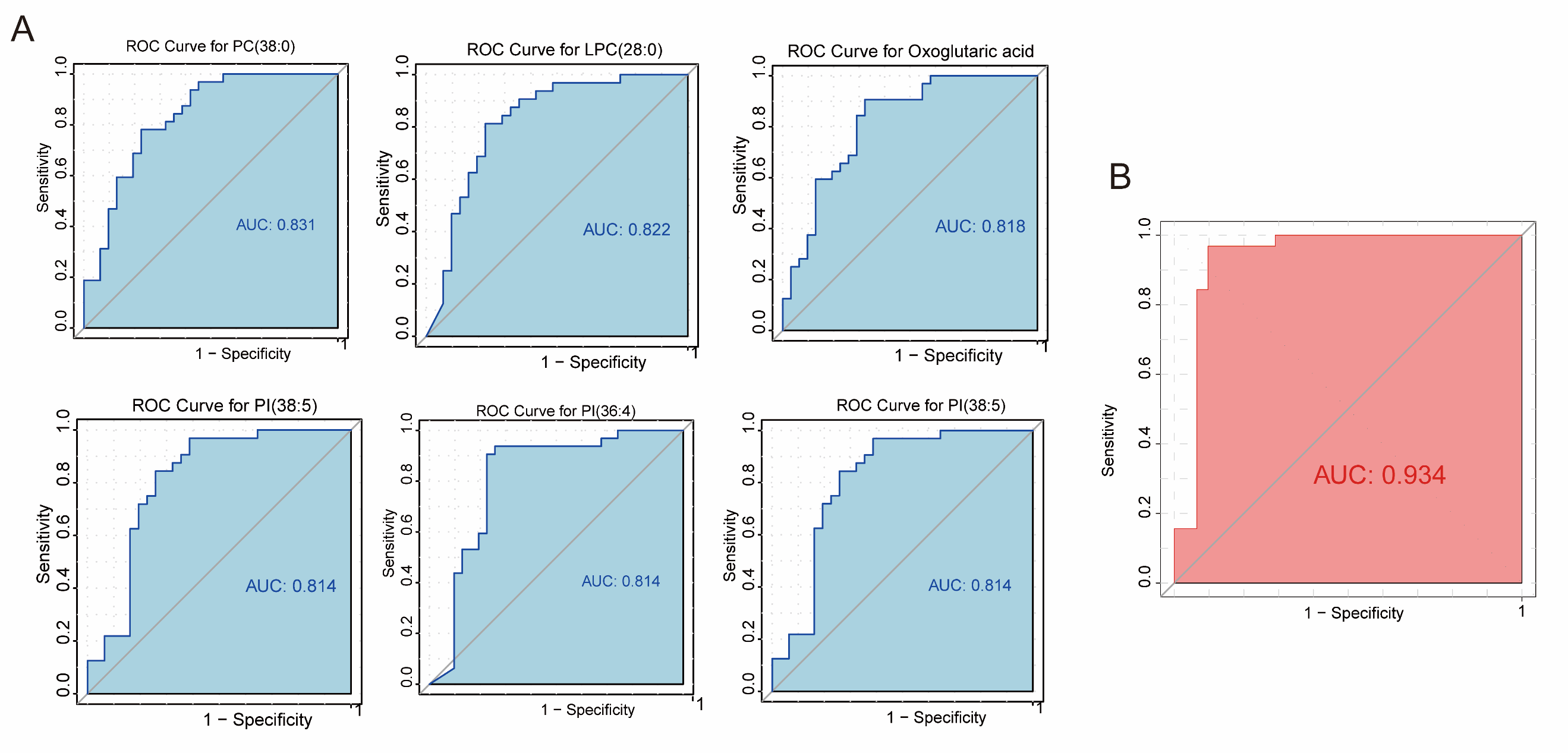


**Supplementary Figure 3. The Potential Diagnostic Capability of IgG4-RD B-cell Biomarkers** (A) ROC curves for the six metabolites with the highest AUC values.
(B) ROC curves of the logistic regression model with 14 metabolites.

ROC, receiver operating characteristic curve; AUC, area under the curve.


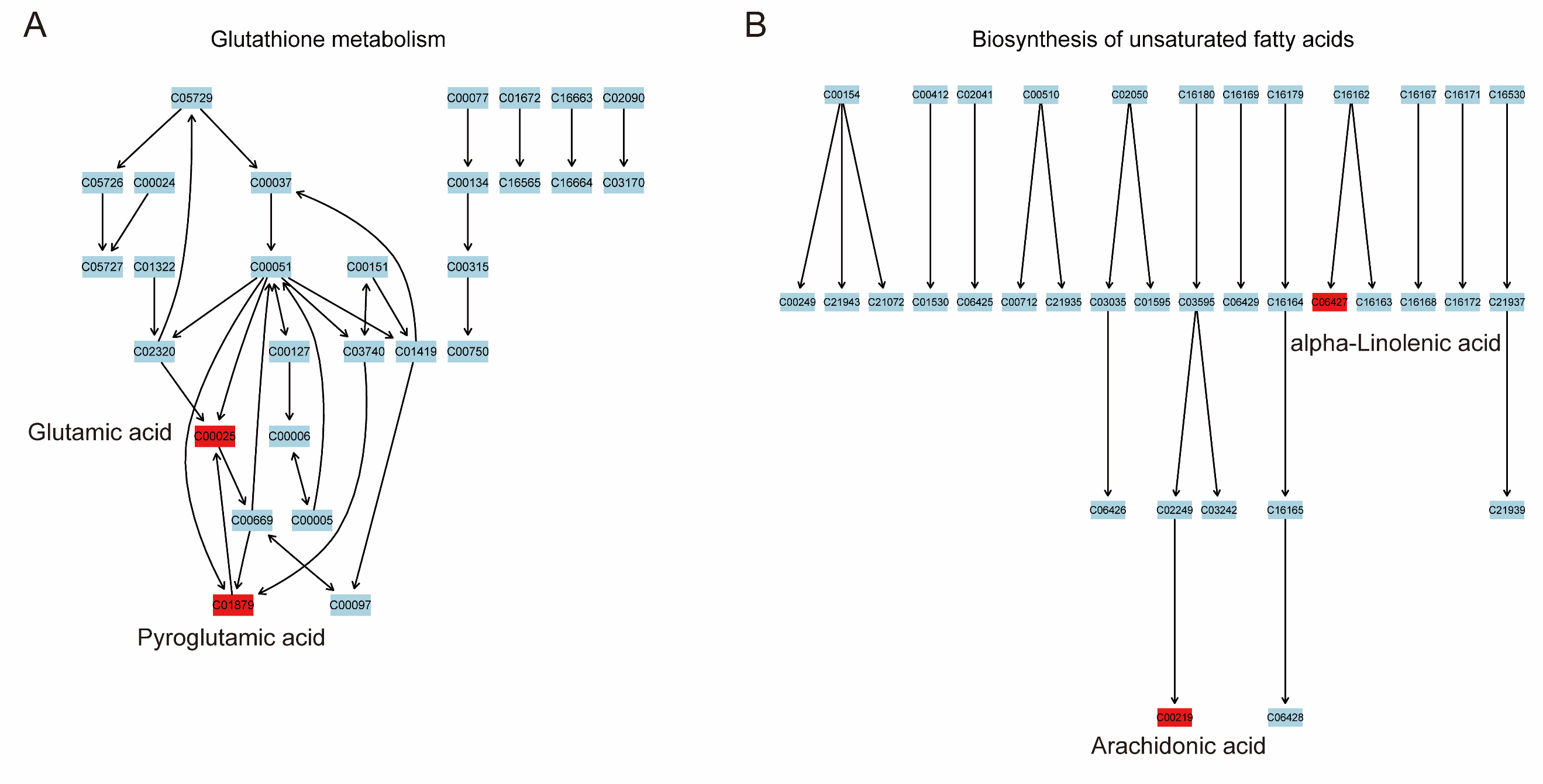


**Supplementary Figure 4. Upregulated metabolic pathways in IgG4-RD B cells. Pathway enrichment analysis was performed with MetaboAnalyst.**

The most significantly upregulated pathways were (A) glutathione metabolism and (B) biosynthesis of unsaturated fatty acids.


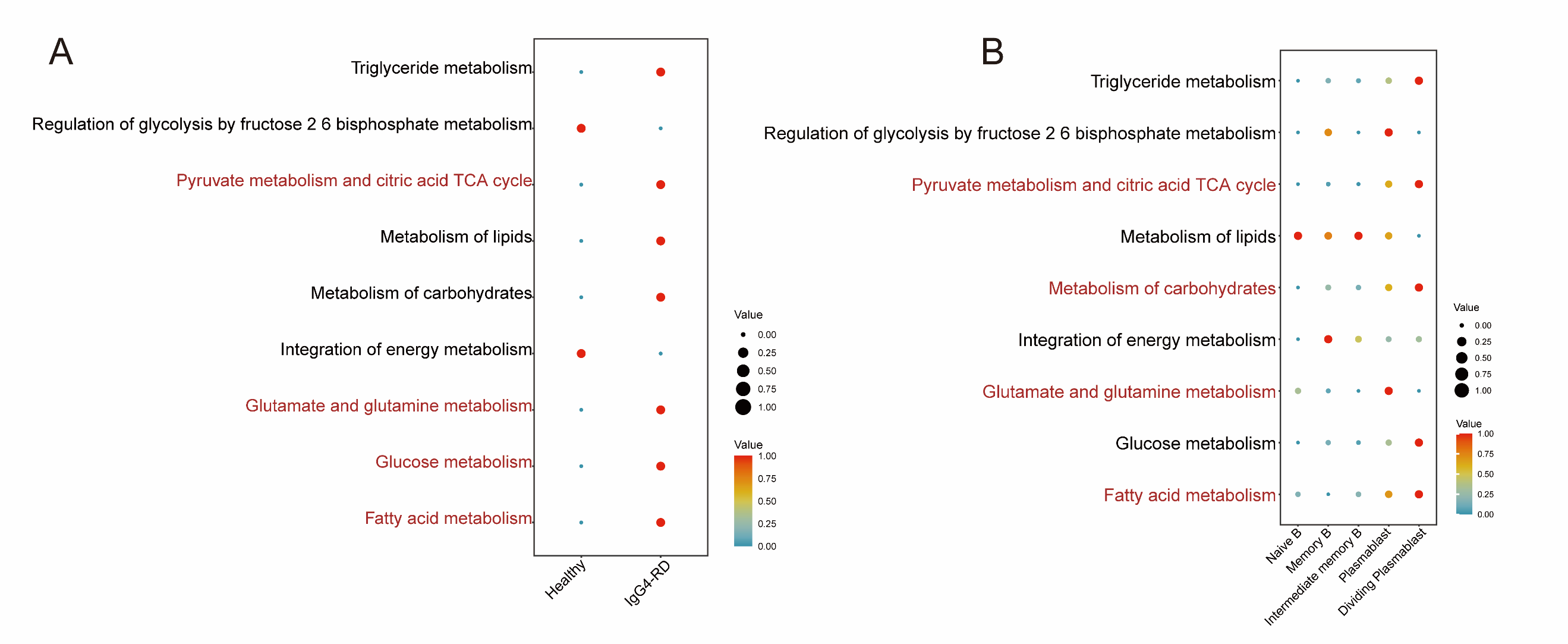


**Supplementary Figure 5. Analysis of metabolic pathway alterations in B cells by single-cell RNA sequencing.**

(A) Transcriptomic differences in metabolic pathways between B cells from IgG4-RD patients and healthy controls.

(B) Heterogeneity of metabolic pathway transcriptomes across different B-cell subsets.


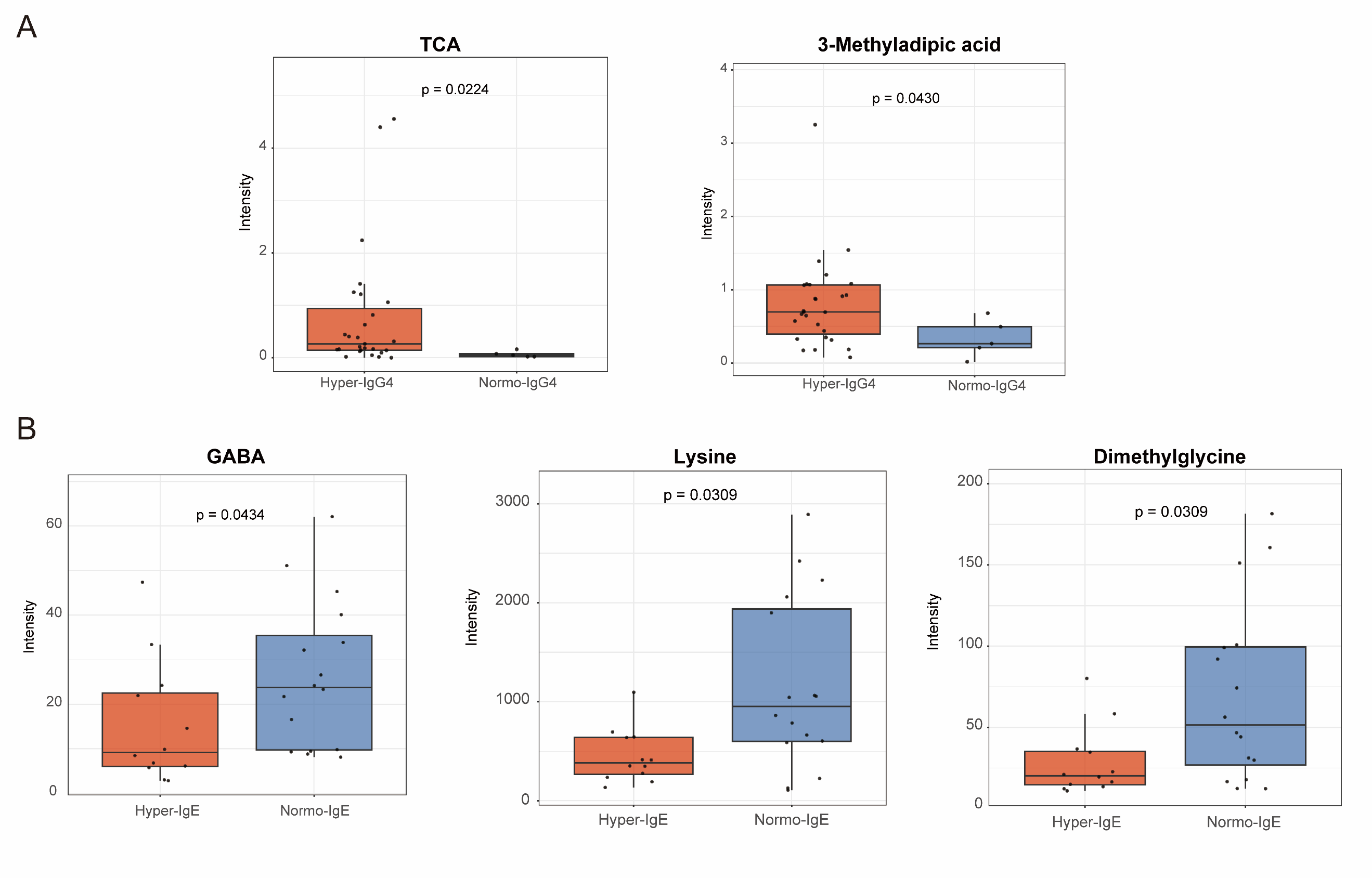


**Supplementary Figure 6. Differential B-cell metabolites in patients stratified by serum IgG4 and IgE status.**

(A) Metabolites differing in B cells from patients with elevated serum IgG4 versus those with normal IgG4 levels.

(B) Metabolites differing in B cells from patients with elevated serum IgE versus those with normal IgE levels.

TCA, taurocholic acid; GABA, γ-aminobutyric acid.

**Supplementary Tables**

**Supplementary table 1: Patient characteristics of the IgG4-RD and control group**

| **ID** | **Sex** | **Age** | **Visit Type** | **Phenotype** | **Treatment Regimen** |
| --- | --- | --- | --- | --- | --- |
| IgG4_1 | Male | 61 | Follow-up | Inflammatory | Pred+MMF |
| IgG4_2 | Female | 56 | New | Fibrotic | N.A. |
| IgG4_3 | Female | 38 | Follow-up | Inflammatory | Pred |
| IgG4_4 | Male | 43 | Follow-up | Inflammatory | Pred+MMF |
| IgG4_5 | Female | 45 | New | Inflammatory | N.A. |
| IgG4_6 | Female | 65 | Follow-up | Fibrotic | Pred+MTX |
| IgG4_7 | Male | 67 | Follow-up | Inflammatory | Pred |
| IgG4_8 | Male | 24 | New | Inflammatory | N.A. |
| IgG4_9 | Male | 47 | Follow-up | Fibrotic | Pred+MTX |
| IgG4_10 | Male | 70 | New | Inflammatory | N.A. |
| IgG4_11 | Male | 56 | New | Inflammatory | N.A. |
| IgG4_12 | Female | 59 | Follow-up | Fibrotic | Pred+MMF |
| IgG4_13 | Male | 49 | New | Fibrotic | N.A. |
| IgG4_14 | Male | 58 | Follow-up | Inflammatory | Pred |
| IgG4_15 | Male | 56 | Follow-up | Inflammatory | Pred |
| IgG4_16 | Male | 53 | Follow-up | Fibrotic | Pred+MMF |
| IgG4_17 | Male | 73 | Follow-up | Inflammatory | Pred |
| IgG4_18 | Male | 42 | Follow-up | Fibrotic | Pred+MMF |
| IgG4_19 | Female | 58 | Follow-up | Inflammatory | Pred+MTX |
| IgG4_20 | Male | 29 | Follow-up | Inflammatory | MTX |
| IgG4_21 | Male | 52 | Follow-up | Inflammatory | Pred+MMF |
| IgG4_22 | Male | 70 | Follow-up | Inflammatory | Pred+MMF |
| IgG4_23 | Male | 55 | New | Inflammatory | N.A. |
| IgG4_24 | Male | 41 | Follow-up | Fibrotic | Pred+iguratimod |
| IgG4_25 | Male | 67 | New | Fibrotic | N.A. |
| IgG4_26 | Male | 72 | Follow-up | Inflammatory | Pred |
| IgG4_27 | Female | 58 | Follow-up | Fibrotic | Pred+MMF |
| IgG4_28 | Male | 67 | New | Fibrotic | N.A. |
| IgG4_29 | Male | 70 | Follow-up | Fibrotic | Pred+MMF |
| IgG4_30 | Male | 46 | Follow-up | Fibrotic | Pred+iguratimod |
| IgG4_31 | Female | 41 | Follow-up | Fibrotic | Pred+CTX |
| IgG4_32 | Male | 54 | Follow-up | Fibrotic | Pred+MMF |
| Con_1 | Female | 52 | N.A. | N.A. | N.A. |
| Con_2 | Male | 55 | N.A. | N.A. | N.A. |
| Con_3 | Male | 62 | N.A. | N.A. | N.A. |
| Con_4 | Female | 58 | N.A. | N.A. | N.A. |
| Con_5 | Female | 52 | N.A. | N.A. | N.A. |
| Con_6 | Male | 56 | N.A. | N.A. | N.A. |
| Con_7 | Male | 57 | N.A. | N.A. | N.A. |
| Con_8 | Male | 51 | N.A. | N.A. | N.A. |
| Con_9 | Male | 58 | N.A. | N.A. | N.A. |
| Con_10 | Female | 61 | N.A. | N.A. | N.A. |
| Con_11 | Male | 57 | N.A. | N.A. | N.A. |
| Con_12 | Male | 55 | N.A. | N.A. | N.A. |
| Con_13 | Female | 61 | N.A. | N.A. | N.A. |
| Con_14 | Male | 63 | N.A. | N.A. | N.A. |
| Con_15 | Female | 59 | N.A. | N.A. | N.A. |
| Con_16 | Male | 55 | N.A. | N.A. | N.A. |
| Con_17 | Female | 55 | N.A. | N.A. | N.A. |
| Con_18 | Female | 68 | N.A. | N.A. | N.A. |
| Con_19 | Male | 60 | N.A. | N.A. | N.A. |
| Con_20 | Female | 73 | N.A. | N.A. | N.A. |
| Con_21 | Male | 60 | N.A. | N.A. | N.A. |
| Con_22 | Male | 60 | N.A. | N.A. | N.A. |
| Con_23 | Female | 58 | N.A. | N.A. | N.A. |
| Con_24 | Male | 63 | N.A. | N.A. | N.A. |
| Con_25 | Male | 57 | N.A. | N.A. | N.A. |
| Con_26 | Male | 60 | N.A. | N.A. | N.A. |
| Con_27 | Male | 64 | N.A. | N.A. | N.A. |
| Con_28 | Male | 63 | N.A. | N.A. | N.A. |
| Con_29 | Male | 23 | N.A. | N.A. | N.A. |
| Con_30 | Female | 52 | N.A. | N.A. | N.A. |
| Con_31 | Male | 24 | N.A. | N.A. | N.A. |

**Supplementary table 2:** **Detailed clinical characteristics of enrolled IgG4-RD patients and healthy controls included in the metabolomic analysis**

|  | **IgG4-RD**  **（n=32）** | **HC**  **(n=31)** | **P** |
| --- | --- | --- | --- |
| **Demography** |  |  |  |
| Sex (male，%) | 24 (75.0%) | 20 (64.5%) | 0.36 |
| Age at onset (years, mean ± S.D.) | 54.5±12.3 | 56.5±9.8 | 0.47 |
| Disease duration (months, median, IQR) | 18.5(8-44) | N.A. |  |
| Affected organ numbers (mean ± S.D.) | 2.2±1.6 | N.A. |  |
| **Serology (mean ± S.D.)** |  |  |  |
| IgG(g/L) | 24.6±16.1 | N.A. |  |
| IgG4(g/L) | 6.4±6.6 | N.A. |  |
| IgE (IU/mL) | 179.5±136.2 | N.A. |  |
| CD19^+^ B cell count (/mL) | (1.3±1.4) * 10^6 | (1.3±1.4) * 10^6 | 0.79 |
| **Organ involvements (n, %)** |  |  |  |
| Lymph node | 12 (37.5%) | N.A. |  |
| Pancreas | 10 (31.3%) | N.A. |  |
| Lacrimal gland/Eyelid | 9 (28.1%) | N.A. |  |
| Lung | 5 (15.6%) | N.A. |  |
| Retroperitoneal tissue | 5 (15.6%) | N.A. |  |
| Salivary gland | 4 (12.5%) | N.A. |  |
| Kidney/Ureter | 3 (9.4%) | N.A. |  |
| Biliary tract | 3 (9.4%) | N.A. |  |
| Prostate | 2 (6.3%) | N.A. |  |
| Breast | 3 (9.4%) | N.A. |  |
| Skull base | 2 (6.3%) | N.A. |  |
| Sinuses, Pharynx | 1 (3.1%) | N.A. |  |
| Thyroid gland | 1 (3.1%) | N.A. |  |
| Gum | 1 (3.1%) | N.A. |  |

**Supplementary table 3. Treatment strategies of IgG4-RD patients included in the metabolomic analysis.**

| **Treatment Strategy** | **Regimen** | **Number of Patients (n, %)** |
| --- | --- | --- |
| GC+IM | Pred+MMF | 10 (31.3) |
| GC | Pred | 6 (18.8) |
| GC+IM | Pred+MTX | 3 (9.4) |
| GC+IM | Pred+iguratimod | 2 (6.3) |
| GC+IM | Pred+CTX | 1 (3.1) |
| IM | MTX | 1 (3.1) |
| Untreated | N.A. | 9 (28.1) |

GC, glucocorticoid; IM, immunosuppressant; Pred, prednisone; MMF, mycophenolate mofetil; MTX, methotrexate; CTX, cyclophosphamide**.**

**Supplementary table 4: Metabolites simultaneously satisfying VIP > 1 and FDR < 0.05.**

| Metabolite | p_value | FC | FDR | VIP | AUC |
| --- | --- | --- | --- | --- | --- |
| PC(38:0) | 6.72E-06 | 0.24242 | 0.000991 | 1.927012 | 0.8306 |
| LPC(28:0) | 1.18E-05 | 0.154686 | 0.000991 | 1.919708 | 0.8216 |
| Oxoglutaric acid | 1.53E-05 | 4.081926 | 0.000991 | 1.886341 | 0.8175 |
| PC(40:3) | 2.22E-05 | 0.271568 | 0.000991 | 1.860399 | 0.8115 |
| PI(36:4) | 1.96E-05 | 0.277939 | 0.000991 | 1.858933 | 0.8135 |
| PC(O-36:0) | 3.61E-05 | 0.279608 | 0.000991 | 1.824627 | 0.8034 |
| PI(38:5) | 1.96E-05 | 0.283179 | 0.000991 | 1.799733 | 0.8135 |
| LPC(18:0) | 5.15E-05 | 0.270642 | 0.000991 | 1.790789 | 0.7974 |
| SM(d16:1/25:0) | 3.20E-05 | 0.33062 | 0.000991 | 1.770964 | 0.8054 |
| Oleylcarnitine | 0.00038 | 0.124742 | 0.002253 | 1.763743 | 0.7611 |
| PC(O-40:4) | 6.50E-05 | 0.310237 | 0.000991 | 1.72956 | 0.7933 |
| SM(d16:1/22:1) | 3.61E-05 | 0.329493 | 0.000991 | 1.727256 | 0.8034 |
| PC(42:5) | 8.19E-05 | 0.323789 | 0.001125 | 1.721823 | 0.7893 |
| PC(40:2) | 6.50E-05 | 0.318714 | 0.000991 | 1.721265 | 0.7933 |
| PC(38:1) | 3.61E-05 | 0.267745 | 0.000991 | 1.716645 | 0.8034 |
| PC(42:9) | 4.85E-05 | 0.237949 | 0.000991 | 1.70716 | 0.7984 |
| PC(O-38:1) | 9.18E-05 | 0.359536 | 0.001184 | 1.704142 | 0.7873 |
| PC(34:4) | 5.46E-05 | 0.255067 | 0.000991 | 1.692092 | 0.7964 |
| PI(40:7) | 0.000144 | 0.302937 | 0.001345 | 1.691831 | 0.7792 |
| SM(d17:1/27:2) | 0.000262 | 0.348027 | 0.001815 | 1.689651 | 0.7681 |
| SM(d18:0/24:0) | 5.79E-05 | 0.295696 | 0.000991 | 1.672888 | 0.7954 |
| Phenylpyruvic acid | 6.88E-05 | 2.86062 | 0.000991 | 1.656243 | 0.7923 |
| PC(42:1) | 4.06E-05 | 0.252371 | 0.000991 | 1.648158 | 0.8014 |
| SM(d16:1/27:2) | 0.000179 | 0.305307 | 0.001491 | 1.645473 | 0.7752 |
| PE(40:3) | 6.47E-05 | 0.332104 | 0.000991 | 1.6393 | 0.7933 |
| LPC(20:0) | 6.50E-05 | 0.258758 | 0.000991 | 1.633054 | 0.7933 |
| PC(O-42:4) | 0.000129 | 0.277531 | 0.001337 | 1.623874 | 0.7812 |
| PC(42:10) | 0.000262 | 0.235605 | 0.001815 | 1.619092 | 0.7681 |
| SM(d16:1/23:0) | 0.000144 | 0.354041 | 0.001345 | 1.609622 | 0.7792 |
| Citric acid | 0.00036 | 2.363928 | 0.002173 | 1.604846 | 0.7621 |
| PC(40:8) | 5.15E-05 | 0.246691 | 0.000991 | 1.599798 | 0.7974 |
| PC(42:2) | 2.14E-05 | 0.190511 | 0.000991 | 1.592791 | 0.812 |
| PE(38:4) | 4.06E-05 | 0.348356 | 0.000991 | 1.591325 | 0.8014 |
| SM(d18:0/26:2) | 0.000235 | 0.374083 | 0.001797 | 1.588095 | 0.7702 |
| Palmitoylcarnitine | 0.001094 | 0.237191 | 0.005114 | 1.586543 | 0.7399 |
| PC(42:8) | 0.000179 | 0.282248 | 0.001491 | 1.583767 | 0.7752 |
| PI(38:3) | 0.000441 | 0.340615 | 0.002514 | 1.58125 | 0.7581 |
| PC(42:3) | 0.000223 | 0.390457 | 0.001775 | 1.574464 | 0.7712 |
| PC(O-38:3) | 0.000342 | 0.365711 | 0.002166 | 1.573213 | 0.7631 |
| Nicotinic acid | 0.002064 | 1.48467 | 0.008301 | 1.572059 | 0.7258 |
| cis-Aconitic acid | 0.0009 | 2.290314 | 0.004259 | 1.568366 | 0.744 |
| Linoleylcarnitine | 0.000152 | 0.09762 | 0.001387 | 1.565612 | 0.7782 |
| Methylcysteine | 0.000492 | 3.431938 | 0.002666 | 1.562138 | 0.756 |
| PC(O-40:5) | 0.000324 | 0.357275 | 0.002127 | 1.561975 | 0.7641 |
| LPC(17:0) | 0.000161 | 0.310158 | 0.001431 | 1.559777 | 0.7772 |
| PI(38:4) | 6.13E-05 | 0.364026 | 0.000991 | 1.559704 | 0.7944 |
| LPC(20:1) | 0.000262 | 0.356893 | 0.001815 | 1.549805 | 0.7681 |
| PC(O-38:6) | 0.00036 | 0.330644 | 0.002173 | 1.546956 | 0.7621 |
| Ketoleucine | 0.000775 | 2.282453 | 0.003973 | 1.543497 | 0.747 |
| SM(d16:0/20:0) | 0.000235 | 0.43372 | 0.001797 | 1.540743 | 0.7702 |
| PC(42:7) | 0.000444 | 0.311065 | 0.002514 | 1.532057 | 0.7581 |
| PE(38:3) | 2.51E-05 | 0.353797 | 0.000991 | 1.530609 | 0.8095 |
| PC(36:0) | 0.000109 | 0.330872 | 0.001271 | 1.523853 | 0.7843 |
| CerPE(16:0) | 0.000775 | 0.197977 | 0.003973 | 1.519549 | 0.745 |
| PC(36:5) | 0.000136 | 0.298285 | 0.001345 | 1.51736 | 0.7802 |
| Cer(d18:1/22:0) | 0.000492 | 0.510345 | 0.002666 | 1.51043 | 0.756 |
| PE(36:2) | 0.000248 | 0.395992 | 0.001815 | 1.509273 | 0.7692 |
| PC(34:3) | 0.00017 | 0.341013 | 0.001477 | 1.504592 | 0.7762 |
| PE(36:4) | 6.89E-05 | 0.372944 | 0.000991 | 1.501354 | 0.7923 |
| PC(O-44:4) | 0.000856 | 0.390595 | 0.004106 | 1.497619 | 0.745 |
| PC(O-34:4) | 0.000109 | 0.263345 | 0.001271 | 1.494551 | 0.7843 |
| PC(40:4) | 0.000308 | 0.382326 | 0.002091 | 1.487974 | 0.7651 |
| SM(d17:1/26:1) | 0.000815 | 0.413506 | 0.00401 | 1.48563 | 0.746 |
| LPC(16:0) | 0.000129 | 0.408974 | 0.001337 | 1.48336 | 0.7812 |
| Tetradecanoylcarnitine | 0.000432 | 0.170175 | 0.002514 | 1.472484 | 0.7586 |
| SM(d16:0/28:1) | 9.71E-05 | 0.339059 | 0.001211 | 1.464781 | 0.7863 |
| SM(d18:2/23:1) | 0.00036 | 0.350783 | 0.002173 | 1.451342 | 0.7621 |
| Gluconolactone | 0.000467 | 5.159582 | 0.002608 | 1.44201 | 0.7571 |
| PC(34:0) | 0.000144 | 0.364741 | 0.001345 | 1.441318 | 0.7792 |
| PE(38:5) | 0.000129 | 0.370979 | 0.001337 | 1.434838 | 0.7812 |
| PC(O-44:6) | 0.001264 | 0.425883 | 0.005563 | 1.419486 | 0.7369 |
| PC(O-38:4) | 0.000738 | 0.354691 | 0.003886 | 1.418799 | 0.748 |
| SM(d17:1/24:1) | 0.00153 | 0.43904 | 0.006502 | 1.416787 | 0.7329 |
| ePE(40:4) | 0.0002 | 0.249311 | 0.001626 | 1.41601 | 0.7732 |
| PC(O-42:5) | 0.002121 | 0.336906 | 0.008351 | 1.415578 | 0.7258 |
| PE(40:5) | 0.000115 | 0.370704 | 0.001304 | 1.415034 | 0.7833 |
| PC(O-34:0) | 0.001148 | 0.413793 | 0.005174 | 1.410157 | 0.7389 |
| ePE(38:4) | 0.001264 | 0.45229 | 0.005563 | 1.400486 | 0.7369 |
| PC(32:0) | 0.000342 | 0.418379 | 0.002166 | 1.392385 | 0.7631 |
| ePE(38:6) | 8.42E-05 | 0.29537 | 0.001125 | 1.391452 | 0.7888 |
| Citramalic acid | 0.001681 | 2.489848 | 0.007064 | 1.38114 | 0.7308 |
| SM(d18:0/15:0) | 0.001423 | 0.369116 | 0.006117 | 1.377673 | 0.7344 |
| PE(40:4) | 0.000324 | 0.379527 | 0.002127 | 1.366351 | 0.7641 |
| PC(40:7) | 0.000856 | 0.338439 | 0.004106 | 1.357932 | 0.745 |
| PE(38:6) | 0.000518 | 0.386318 | 0.002766 | 1.354085 | 0.755 |
| PE(36:3) | 0.001148 | 0.429616 | 0.005174 | 1.347973 | 0.7389 |
| Dimethylglycine | 0.000815 | 1.636919 | 0.00401 | 1.345524 | 0.746 |
| Asparagine | 0.003185 | 0.459763 | 0.011144 | 1.341281 | 0.7167 |
| PE(40:6) | 0.001934 | 0.441963 | 0.007948 | 1.337892 | 0.7278 |
| 3-Methyl-2-oxopentanoic acid | 0.007193 | 2.345034 | 0.020381 | 1.33057 | 0.6976 |
| Cer(d18:1/24:1) | 0.001148 | 0.594961 | 0.005174 | 1.326341 | 0.7389 |
| PC(44:11) | 0.002788 | 0.449653 | 0.010222 | 1.324124 | 0.7198 |
| Methylmalonic acid | 0.002788 | 1.796267 | 0.010222 | 1.32319 | 0.7198 |
| PC(O-34:3) | 0.002325 | 0.439164 | 0.008965 | 1.309792 | 0.7238 |
| PC(O-30:1) | 0.003641 | 0.44661 | 0.012378 | 1.307341 | 0.7137 |
| Succinic acid | 0.004724 | 1.784661 | 0.014974 | 1.295564 | 0.7077 |
| SM(d16:2/23:0) | 0.004334 | 0.478273 | 0.014096 | 1.291528 | 0.7097 |
| Oxalic acid | 0.002026 | 2.073544 | 0.008234 | 1.275245 | 0.7268 |
| PC(44:12) | 0.003333 | 0.467541 | 0.011542 | 1.273161 | 0.7157 |
| ePE(36:4) | 0.000248 | 0.350695 | 0.001815 | 1.261052 | 0.7692 |
| PC(30:0) | 0.000815 | 0.442627 | 0.00401 | 1.259842 | 0.746 |
| SM(d18:0/14:0) | 0.007193 | 0.482522 | 0.020381 | 1.254758 | 0.6976 |
| LPC(20:2) | 0.002221 | 0.4958 | 0.008653 | 1.253223 | 0.7248 |
| 1-Methylhistidine | 0.002547 | 1.778247 | 0.009621 | 1.252289 | 0.7218 |
| SM(d19:1/16:0) | 0.004334 | 0.493829 | 0.014096 | 1.24735 | 0.7097 |
| PC(32:2) | 0.001391 | 0.413243 | 0.006051 | 1.245983 | 0.7349 |
| Carnosine | 0.00415 | 0.536366 | 0.013737 | 1.245216 | 0.7107 |
| PC(O-40:6) | 0.004931 | 0.425819 | 0.015369 | 1.245 | 0.7067 |
| PC(O-44:5) | 0.006902 | 0.499143 | 0.020012 | 1.244661 | 0.6986 |
| SM(d16:1/18:1) | 0.002434 | 0.466378 | 0.009288 | 1.241911 | 0.7228 |
| PC(O-34:2) | 0.003804 | 0.473407 | 0.012702 | 1.2331 | 0.7127 |
| SM(d18:0/16:0) | 0.003049 | 0.486452 | 0.010966 | 1.214245 | 0.7177 |
| SM(d18:1/19:0) | 0.003188 | 0.479588 | 0.011144 | 1.213933 | 0.7167 |
| PC(O-40:2) | 0.005842 | 0.505399 | 0.017621 | 1.208172 | 0.7026 |
| PC(O-36:4) | 0.003188 | 0.459086 | 0.011144 | 1.208086 | 0.7167 |
| SM(d18:1/25:0) | 0.005369 | 0.531912 | 0.01646 | 1.202532 | 0.7046 |
| SM(d18:2/14:0) | 0.009944 | 0.538081 | 0.026755 | 1.202419 | 0.6895 |
| 3-Hydroxylisovalerylcarnitine | 0.002916 | 0.430477 | 0.010587 | 1.201919 | 0.7188 |
| PC(O-38:5) | 0.003804 | 0.455544 | 0.012702 | 1.187016 | 0.7127 |
| SM(d16:1/16:0) | 0.004931 | 0.500263 | 0.015369 | 1.181968 | 0.7067 |
| Homocitrulline | 0.002665 | 2.436675 | 0.009966 | 1.173408 | 0.7208 |
| 2-Methylbutyroylcarnitine | 0.007342 | 0.54468 | 0.020646 | 1.161554 | 0.6971 |
| PC(28:1) | 0.007808 | 0.514129 | 0.02163 | 1.158942 | 0.6956 |
| PI(36:2) | 0.010346 | 0.512196 | 0.027444 | 1.150049 | 0.6885 |
| PC(O-36:1) | 0.004724 | 0.518474 | 0.014974 | 1.147834 | 0.7077 |
| Homoserine | 0.005369 | 1.812704 | 0.01646 | 1.138751 | 0.7046 |
| Phthalic acid | 0.007193 | 0.476932 | 0.020381 | 1.129672 | 0.6976 |
| PC(O-40:1) | 0.012585 | 0.528501 | 0.032461 | 1.122722 | 0.6835 |
| ePE(38:5) | 0.002121 | 0.414251 | 0.008351 | 1.119996 | 0.7258 |
| alpha-Hydroxyisobutyric acid | 0.006093 | 1.915211 | 0.018084 | 1.111013 | 0.7016 |
| Arachidonic acid | 0.006902 | 1.899554 | 0.020012 | 1.095732 | 0.6986 |
| PC(O-38:2) | 0.013081 | 0.570747 | 0.03351 | 1.093537 | 0.6825 |
| Ribose 5-phosphate | 0.008818 | 0.528847 | 0.024073 | 1.090561 | 0.6925 |
| PE(40:7) | 0.009365 | 0.423218 | 0.02538 | 1.069111 | 0.691 |
| Tartaric acid | 0.014126 | 2.19426 | 0.035457 | 1.056661 | 0.6804 |
| Palmitoleic acid | 0.017065 | 3.485409 | 0.041714 | 1.053168 | 0.6754 |
| PC(O-32:1) | 0.006622 | 0.568145 | 0.019502 | 1.052777 | 0.6996 |
| Dodecanoylcarnitine | 0.008467 | 0.329122 | 0.023286 | 1.051454 | 0.6935 |
| PC(O-44:3) | 0.003641 | 0.450306 | 0.012378 | 1.042993 | 0.7137 |
| PC(O-34:1) | 0.010763 | 0.536188 | 0.028151 | 1.039018 | 0.6875 |
| PE(32:1) | 0.013595 | 5.035544 | 0.034354 | 1.03549 | 0.6815 |
| PC(O-40:3) | 0.019075 | 0.54149 | 0.046026 | 1.034704 | 0.6724 |
| Pyroglutamic acid | 0.007808 | 1.433864 | 0.02163 | 1.030105 | 0.6956 |
| alpha-Linolenic acid | 0.014397 | 0.579693 | 0.035896 | 1.025426 | 0.6799 |

**Supplementary Table 5: Summary of clinical characteristics of enrolled IgG4-RD patients and healthy controls included in the flow cytometry analysis.**

|  | **IgG4-RD**  **（n=20）** | **HC**  **(n=6)** |
| --- | --- | --- |
| **Demography** |  |  |
| Sex (male，%) | 15(75.0%) | 4 (66.7%) |
| Age at onset (years, mean ± S.D.) | 52.6±13.4 | 53.5±14.1 |
| Affected organ numbers (mean ± S.D.) | 1.9±1.6 | N.A. |
| Treatment-naive | 0 | N.A. |
| **Serology (mean ± S.D.)** |  |  |
| IgG(g/L) | 21.7±12.0 | N.A. |
| IgG4(g/L) | 16.0±22.0 | N.A. |
| IgE (IU/mL) | 592.9±744.7 | N.A. |
| **Organ involvements (n%)** |  |  |
| Lacrimal gland/Eyelid | 8 (40%) | N.A. |
| Pancreas | 7 (35%) | N.A. |
| Lymph node | 6 (30%) | N.A. |
| Salivary gland | 5 (25%) | N.A. |
| Lung | 2 (10%) | N.A. |
| Kidney/Adrenal gland | 2 (10%) | N.A. |
| Biliary tract | 2 (10%) | N.A. |
| Retroperitoneal tissue | 1 (5%) | N.A. |
| Prostate | 1 (5%) | N.A. |
| Sinuses, Pharynx | 1 (5%) | N.A. |

**Supplementary Table 6. Treatment strategies of IgG4-RD patients enrolled in the flow cytometry analysis.**

| **Treatment Strategy** | **Regimen** | **Number of Patients (n, %)** |
| --- | --- | --- |
| GC+IM | Pred+MMF | 12 (60.0) |
| GC | Pred | 5 (25.0) |
| GC+IM | Pred+MTX | 1 (5.0) |
| GC+IM | Pred+iguratimod | 1 (5.0) |
| GC+IM | Pred+CTX | 1 (5.0) |

GC, glucocorticoid; IM, immunosuppressant; Pred, prednisone; MMF, mycophenolate mofetil; MTX, methotrexate; CTX, cyclophosphamide**.**

**Supplementary Table 7. Comparison of clinical characteristics between IgG4-RD patients with fibrotic and inflammatory phenotypes.**

|  | **Inflammatory phenotype**  **（n=17）** | **Fibrotic phenotype**  **(n=15)** | **P** |
| --- | --- | --- | --- |
| **Demography** |  |  |  |
| Sex (male，%) | 14 (82.4%) | 10 (66.7%) | 0.31 |
| Age at onset (years, mean ± S.D.) | 54.5±14.2 | 54.3±9.6 | 0.97 |
| Treatment-naive | 5 (29.4%) | 4 (26.7%) | 0.86 |
| Affected organ numbers (mean ± S.D.) | 2.4±1.9 | 2.0±1.3 | 0.56 |
| IgG4-RI | 3.0±2.3 | 2.4±1.5 | 0.47 |
| **Serology (mean ± S.D.)** |  |  |  |
| IgG(g/L) | 17.9±8.9 | 27.0±19.2 | 0.16 |
| IgG4(g/L) | 5.5±4.2 | 7.5±8.4 | 0.41 |
| IgE (IU/mL) | 189.9±131.2 | 167.5±140.8 | 0.68 |
| **Main organs involved (n)** |  |  |  |
| Orbital pseudotumour | 0 | 2 |  |
| Skull base | 0 | 2 |  |
| Retroperitoneal tissue | 0 | 5 |  |
| Mediastinitis | 0 | 2 |  |
| Pancreas | 10 | 0 |  |
| Lacrimal gland | 7 | 0 |  |
| Salivary gland | 4 | 0 |  |
| Biliary tract | 3 | 0 |  |
| **Regimen (n)** |  |  |  |
| Pred+MMF | 4 | 6 |  |
| Pred | 6 | 0 |  |
| Untreated | 5 | 4 |  |
| Pred+MTX | 1 | 2 |  |
| MTX | 1 | 0 |  |
| Pred+iguratimod | 0 | 2 |  |
| Pred+CTX | 0 | 1 |  |

Pred, prednisone; MMF, mycophenolate mofetil; MTX, methotrexate; CTX, cyclophosphamide.
